# Evaluating the role of faecal calprotectin in older adults: a retrospective observational study

**DOI:** 10.3399/BJGP.2025.0169

**Published:** 2025-11-18

**Authors:** Robert W Perry, Peter FG Foulser, David Zhang, Pablo Martinez Perez, Shakira Taylor, Angelica Sharma, Mithun Kumaran, Sharmili Balarajah, Shiva T Radhakrishnan, Rohan Sundramoorthi, Carmen MY Chung, David Mummery, James L Alexander, Azeem Majeed, Lucy C Hicks, Horace RT Williams

**Affiliations:** 1 Department of Metabolism, Digestion and Reproduction, Faculty of Medicine, Imperial College London, London, UK; 2 Departments of Gastroenterology and Hepatology, St Mary’s Hospital, Imperial College Healthcare NHS Trust, London, UK; 3 Northwick Park Hospital, London North West University Healthcare NHS Trust, Harrow, UK; 4 Department of Primary Care and Public Health, Imperial College London, London, UK; 5 St Mark’s Hospital, Central Middlesex, London, UK

**Keywords:** faecal calprotectin, faecal immunochemical test, gastroenterology, inflammatory bowel diseases, primary care

## Abstract

**Background:**

Faecal calprotectin (FC) is a marker of gastrointestinal (GI) inflammation that is widely used in primary care to identify patients who need to be referred for endoscopic assessment. Most guidelines advise against its use in older adults because of higher rates of colorectal cancer (CRC) in this group, and many studies include only younger adults, even though FC is still used in older adults.

**Aim:**

To evaluate FC performance in identifying inflammatory bowel disease (IBD) and significant organic pathology in adults aged ≥50 years, to help inform its use and interpretation in primary care. Where available, faecal immunochemical test (FIT) data were also analysed.

**Design and setting:**

A retrospective observational study of patients who were referred from primary care to secondary care for colonoscopy at Imperial College Healthcare NHS Trust.

**Method:**

Patients who had undergone FC testing, followed by a colonoscopy, were recruited and grouped into younger adults (aged 18–49 years) and older adults (aged ≥50 years). Data were then collected using electronic health records to allow for analysis of FC diagnostic performance.

**Results:**

In total, 669 patients were included: 423 aged 18–49 years and 246 aged ≥50 years. There were no significant differences in FC levels between the groups (median FC level: 71 µg/g versus 85.5 µg/g respectively, *P* = 0.29). An FC level of >50 µg/g showed a high sensitivity for inflammatory bowel disease (IBD) in both groups (94.1% in those aged 18–49 years and 93.8% in those aged ≥50 years), but the positive predictive value was low — particularly in those aged ≥50 years (12.8%) versus the younger cohort (20.9%). An FC level of >50 µg/g outperformed FIT (threshold 10 µg/g) for the diagnosis of IBD and organic pathology in both groups. However, in patients aged ≥50 years, FIT outperformed FC at 150 µg/g for the diagnosis of organic pathology, including CRC.

**Conclusion:**

These data show that FC remains a sensitive test in older adults. FC may have a role as a ‘rule-out’ test in adults aged ≥50 years who have lower GI symptoms and a negative FIT, when CRC is not suspected.

## How this fits in

There is considerable uncertainty surrounding the use of faecal calprotectin (FC) as a diagnostic test in older adults, with varying suggestions in guidelines and a lack of data in the wider literature. This study investigated the performance of FC in older adults (aged ≥50 years) compared with a younger cohort, with a view to guiding correct use of it in a primary care setting. The findings suggest that FC is a sensitive test for inflammatory bowel disease and organic gastrointestinal pathology in both groups; however, concerns remain over its positive predictive value and specificity — particularly in older adults — and it should not be used if colorectal cancer is suspected.

## Introduction

Gastrointestinal (GI) symptoms are common, accounting for ~10% of presentations to general practice.^1,2^ In primary care, it is essential to differentiate between significant conditions (such as inflammatory bowel disease [IBD]), for which urgent referral for further investigation is required, and functional disorders (such as irritable bowel syndrome [IBS]), for which first-line management can be delivered in primary care. Faecal calprotectin (FC) is a well-established marker of GI inflammation and, therefore, widely used to identify patients who require referral for further investigation, often through endoscopic assessment.^3^ Its low cost and stability at room temperature make it suitable for use in a wide variety of healthcare settings.^4^ However, there is uncertainty surrounding the role of FC in older adults due to a higher likelihood of colorectal cancer (CRC) in patients with GI symptoms in this group. Clinical guidelines often advocate a much lower threshold for endoscopic investigation with increasing age and, as such, studies of FC have often excluded older adults.^5,6^ In addition, the clinical guidelines available to primary care clinicians vary in their advice surrounding FC use and age.^7^ Despite this, data show that FC tests continue to be performed in older adults, so evaluation of its performance is important.^8^

Many studies have shown the efficacy of FC in differentiating IBD from functional GI conditions, such as IBS, in young patients, including those with alarm symptoms.^5,6,9^ Most guidelines, however, only recommend use of FC in patients in whom CRC is not suspected because of data suggesting FC lacks sensitivity for CRC.^10,11^ Guidelines published by the National Institute for Health and Care Excellence (NICE) do not suggest a firm age threshold above which FC should not be used, but do state that age in the presence of certain GI symptoms confers a risk of cancer that requires a faecal immunochemical test (FIT) or urgent endoscopy, rather than FC testing.^12^ The British Society of Gastroenterology (BSG) guidelines for investigation of chronic diarrhoea do give a clearer age cut-off: they advise using FC to exclude colonic inflammation in those with suspected IBS aged <40 years.^13^ The NICE-endorsed York Faecal Calprotectin Care Pathway recommends an age cut-off of 60 years.^14^

Typically, an FC level of 50 µg/g has been used as the threshold of positivity, either prompting repeat testing or referral for investigation. However, at this cut-off, FC often results in unnecessary investigation and, therefore, various alternative FC thresholds ranging from 50 µg/g to 250 µg/g have been proposed to optimise sensitivity and specificity.^7,15^ Another approach is the retesting of borderline elevated results, which was suggested in the York Faecal Calprotectin Care Pathway.^14^ However, whether similar cut-offs and approaches apply to older adults with suspected inflammatory pathology requires more investigation. A 2024 study by Gallo *et al* ^16^ showed that similar FC thresholds can be used in adults aged >65 years and in younger adults to identify inflammatory pathology, with a high specificity. Other studies in healthy adults have suggested that FC levels may increase with age, highlighting the need for further research.^17^

This study aimed to evaluate the performance of FC in identifying IBD and significant organic pathology in adults aged ≥50 years to help inform the use of FC in primary care. The efficacy of FIT relative to FC in both age cohorts was also assessed, given both the widespread use of FIT in patients presenting with lower GI symptoms and frequent use of it in clinical practice alongside FC.

## Method

### Study design and sample recruitment

This retrospective single-centre study evaluated adults aged ≥18 years who had undergone FC testing in primary care or a hospital setting for diagnostic purposes. Patients were consecutively recruited over a 6-month period. Those who had undergone FC testing and a subsequent colonoscopy performed at Imperial College Healthcare NHS Trust between May and October 2021 were identified through electronic health records. Patients with existing IBD were excluded.

For analysis, patients were grouped into:

- younger adults, defined as those aged 18–49 years; and
- older adults, defined as those ≥50 years.

An age of 50 years was used to define an ‘older adult’ because this cut-off was being used in the NICE guidelines as a threshold above which the GI symptoms of abdominal pain, rectal bleeding, and weight loss are individually considered to indicate there being a high risk of cancer.

### Data collection

Using electronic health records and pathology requests, data were collected to determine presenting symptoms and indications for testing. Data were anonymised prior to analysis and kept in a secure electronic database. Blood results (haemoglobin, C-reactive protein [CRP]), FC, FIT, and all endoscopic, radiological, and pathology reports were reviewed. All patients were allocated a final diagnosis based on the available clinical documentation. These diagnoses were:

- normal;
- IBD;
- IBS or other functional GI disorder;
- CRC;
- other significant GI diagnoses (polyps measuring ≥10 mm, diverticulitis, ischaemic colitis, microscopic colitis); and
- other non-significant GI diagnoses (for which a diagnosis had been identified following investigation, but was deemed not clinically significant — these included polyps measuring <10 mm, uncomplicated diverticular disease, haemorrhoids).

The diagnoses were then grouped into the following categories for analysis:

- organic GI pathology (IBD, CRC, other significant GI diagnoses); or
- non-organic GI pathology (normal, IBS or other functional GI disorders, and other non-significant GI diagnoses).

If the diagnosis was not clear from the available documentation, the case was reviewed by two clinicians to determine the most accurate diagnosis.

### Statistical analysis

Statistical analysis was performed in GraphPad Prism (version 10.4.0). The performance of FC was analysed in both cohorts, calculating the sensitivity, specificity, positive predictive value (PPV), and negative predictive value (NPV) at a threshold of 50 µg/g and 150 µg/g for the diagnosis of IBD compared with non-organic GI pathology. The same analyses were then also performed comparing the ability of FC to distinguish between all organic GI pathology and non-organic pathology. An FC threshold of 50 µg/g was used as it is the threshold recommended in the NICE guidelines, and 150 µg/g was used as it is the threshold used by local pathways, above which immediate referral — rather than repeat testing — is suggested.^11^ Where available, FIT results underwent the same statistical analysis, using a threshold for a positive FIT of 10 µg/g, as recommended in BSG guidelines on investigating suspected CRC.^18^

The area under the receiver operating characteristic (ROC) curve was calculated for the ability of FC to diagnose IBD and significant GI pathology in both age groups. Youden’s index was used to calculate the optimal cut-off.

Unpaired *t*-tests were used to analyse differences in FC levels between the two age groups. Chi-squared (χ^2^) and Fisher’s exact testing were used to assess the relationship between presenting symptoms, FIT testing, CRP levels, FC levels, haemoglobin levels, and a diagnosis of IBD and organic GI pathology. Any of these variables that were found to be predictive of a diagnosis of organic GI pathology were then entered into a multiple logistic regression model.

## Results

### Demographic and baseline data

In total, 669 patients met the eligibility criteria. Of these, 423 were aged 18–49 years (median 33 years, interquartile range [IQR] 27–42 years, 60.0% female) and 246 were aged ≥50 years (median 58 years, IQR 53–68 years, 54.1% female) (Table 1). Of the groups aged 18–49 years and ≥50 years, 349 (82.5%) and 187 (76.0%) underwent FC testing in primary care respectively (data not shown). The median time from FC to endoscopy was 13 weeks (IQR 4–22 weeks) in the cohort aged 18–49 years, and 7 weeks (IQR 3–16) in the cohort aged ≥50 years (*P*<0.001).

**Table 1.** Summary of key demographic details, indications for FC testing, ultimate diagnosis, and FC levels for patients included in the study

	Cohort			
	Total, *n* = 669	18–49 years, *n* = 423	≥50 years, *n* = 246	*P*-value (18–49 years versus >50 years)
**Demographic and clinical features**				
Median age, years (IQR)	43 (31–55)	33 (27–42)	58 (53–68)	
Female, *n* (%)	387 (57.8)	254 (60.0)	133 (54.1)	0.14
Haemoglobin g/L (IQR)	136 (126–147)	137 (126–148)	135 (125–144)	0.26
C-reactive protein mg/L (IQR)	1.6 (0.6–6.4)	1.4 (0.5–6.1)	2.1 (0.8–8.2)	0.52
Calprotectin µg/g, median (IQR)	77 (20–234)	71 (20–215)	85.5 (20–250)	0.29
Calprotectin ≥50 µg/g, *n* (%)	395 (59.0)	250 (59.1)	145 (58.9)	0.71
FIT performed, *n* (%)	401 (59.9)	249 (58.9)	152 (61.8)	0.30
FIT µg/g, median (IQR)	6.0 (6.0–15.0)	6.0 (6.0–14.0)	6.0 (6.0–23.0)	0.51
FIT ≥10 µg/g, *n* (%)	133 (33.2)	77 (30.9)	56 (36.8)	0.22
**Indications to undergo FC testing**				
Abdominal pain, *n* (%)	118 (17.6)	72 (17.0)	46 (18.7)	0.6
Diarrhoea, *n* (%)	201 (30.0)	116 (27.4)	85 (34.6)	0.055
Change in bowel habit, *n* (%)	154 (23.0)	103 (24.3)	51 (20.7)	0.27
Per rectum bleeding, *n* (%)	127 (19.0)	98 (23.2)	29 (11.8)	**<0.001**
Other, *n* (%)	69 (10.3)	34 (8.0)	35 (14.2)	**0.013**
**Main final diagnosis**				
Normal, *n* (%)	306 (45.7)	207 (48.9)	99 (40.2)	**0.03**
IBD, *n* (%)	67 (10.0)	51 (12.1)	16 (6.5)	**0.02**
IBS (or other functional), *n* (%)	97 (14.5)	84 (19.9)	13 (5.3)	**<0.001**
Colorectal cancer, *n* (%)	7 (1.0)	2 (0.5)	5 (2.0)	0.11
Other significant diagnosis, *n* (%)	62 (9.3)	28 (6.6)	34 (13.8)	**0.003**
Other non-significant diagnosis, *n* (%)	130 (19.4)	51 (12.1)	79 (32.1)	**<0.001**
Median time from FC to colonoscopy in weeks, *n* (IQR)	10 (4–20)	13 (4–22)	7 (3–16)	**<0.001**
**Median FC level for each diagnosis**				
Diagnosed normal, median (IQR)	58 (20–157)	58 (20–157)	59 (20–166)	0.29
IBD, median (IQR)	612 (309–2126)	648 (309–3043)	506 (248–1939)	0.85
IBS (or other functional disorder), median (IQR)	56 (20–139)	53.5 (20–114)	206 (63–551)	**0.007**
Colorectal cancer, median (IQR)	253 (107–439)	346 (253–439)	217 (99–493)	0.73
Other significant diagnosis, median (IQR)	151 (20–736)	131 (20–1183)	190 (20–675)	0.27
Other non-significant diagnosis, median (IQR)	54 (24–163)	54 (20–168)	56 (25–166)	0.39

P-values in bold signify statistical significance (<0.05). FC = faecal calprotectin. FIT = faecal immunochemical test. IBD = inflammatory bowel disease. IBS = irritable bowel syndrome. IQR = interquartile range.

Diarrhoea was the most common indication for testing in both groups (116 [27.4%] of the cohort aged 18–49 years, and 85 [34.6%] of the cohort aged ≥50 years, *P*=0.055) (Table 1). Of the younger and older cohorts, 51 (12.1%) and 16 (6.5%) patients were diagnosed with IBD respectively (*P* = 0.02) (Table 1). Two (0.5%) of the 423 patients aged 18–49 years were diagnosed with CRC, compared with five (2.0%) of the 246 patients aged ≥50 years (*P* = 0.11) (Table 1). Older adults were also significantly more likely to be diagnosed with other significant GI diagnoses (*P* = 0.003) or other non-significant GI diagnoses (*P*<0.001).

There were no significant differences in FC levels between the two age groups (median FC 71 µg/g [IQR 20–215] in those aged 18–49 years versus 85.5 µg/g [IQR 20–250] in those aged ≥50 years, *P* = 0.29) (Table 1). Additionally, there was no difference between the two groups in terms of the number of patients with FC ≥50 µg/g (59.1% in those aged 18–49 years versus, 58.9% in those aged ≥50 years, *P* = 0.71) (Table 1). In those diagnosed with functional GI disease, FC was significantly elevated in the cohort aged ≥50 years compared with those aged 18–49 years (median 206 µg/g versus 53.5 µg/g respectively, *P* = 0.007), but this was not the case across the other diagnostic categories (Tables 1 and 2).

### FC diagnostic performance

In both cohorts, median FC was significantly higher in those diagnosed with IBD versus those diagnosed with non-organic GI pathology: 648 µg/g (Table 1) versus 55.5 µg/g (*P*<0.001) in the cohort aged 18–49 years, and 506 µg/g (Table 1) versus 61 µg/g (*P*<0.001) in the cohort aged ≥50 years (data not shown).

In the cohort aged 18–49 years, at a threshold of 50 µg/g, FC had a sensitivity of 94.1% (95% confidence interval [CI] = 84.1 to 98.4) for diagnosing IBD versus non-organic GI pathology, and a specificity of 46.8% (95% CI = 41.6 to 52.1); the PPV was 20.9% (95% CI = 16.1 to 26.6), and the NPV was 98.2% (95% CI = 94.7 to 99.5) (Table 2). In the cohort aged ≥50 years, sensitivity and specificity were similar to the younger cohort (93.8% [95% CI = 71.7 to 99.7] and 46.6% [95% CI = 39.7 to 53.7]) respectively, with an NPV of 98.9% (95% CI = 93.8 to 99.9); the PPV was, however, low at 12.8% (95% CI = 7.9 to 20.1) in the cohort of older adults (Table 2).

**Table 2.** Summary of FC and FIT diagnostic performance for the diagnosis of both IBD and organic GI pathology at varying thresholds

Age category	Sensitivity, % (95% CI)	Specificity, % (95% CI)	PPV, % (95% CI)	NPV, % (95% CI)	Likelihood ratio
**IBD versus non-organic GI pathology (FC threshold 50 µg/g)**					
≥50 years	93.8 (71.7 to 99.7)	46.6 (39.7 to 53.7)	12.8 (7.9 to 20.1)	98.9 (93.8 to 99.9)	1.76
18–49 years	94.1 (84.1 to 98.4)	46.8 (41.6 to 52.1)	20.9 (16.1 to 26.6)	98.2 (94.7 to 99.5)	1.77
**IBD versus non-organic GI pathology (FC threshold 150 µg/g)**					
≥50 years	81.3 (56.9 to 93.4)	70.2 (63.3 to 76.2)	18.6 (11.2 to 29.2)	97.8 (93.8 to 99.4)	2.72
18–49 years	86.3 (74.3 to 93.2)	75.7 (70.9 to 80.0)	34.7 (26.9 to 43.3)	97.4 (94.7 to 98.7)	3.56
**Organic versus non-organic GI pathology (FC threshold 50 µg/g)**					
≥50 years	78.2 (65.6 to 87.1)	46.6 (39.7 to 53.7)	29.7 (22.8 to 37.5)	88.1 (80.4 to 93.1)	1.46
18–49 years	84.0 (74.5 to 90.4)	46.8 (41.6 to 52.1)	27.2 (22.1 to 33.0)	92.5 (87.6 to 95.6)	1.58
**Organic versus non-organic GI pathology (FC threshold 150 µg/g)**					
≥50 years	60.0 (46.8 to 71.9)	70.2 (63.3 to 76.2)	36.7 (27.5 to 47.0)	85.9 (79.6 to 90.5)	2.01
18–49 years	74.1 (63.6 to 82.4)	75.7 (70.9 to 80.0)	42.0 (34.2 to 50.2)	92.5 (88.8 to 95.0)	5.05
**FIT testing (using threshold of 10 µg/g) for organic versus non-organic GI pathology**					
≥50 years	69 (50.8 to 82.7)	70.7 (62.2 to 78.1)	35.7 (24.5 to 48.8)	90.6 (83.1 to 95.0)	2.36
18–49 years	68.4 (52.4 to 80.9)	75.8 (69.6 to 81.1)	33.8 (24.2 to 44.9)	93.0 (88.2 to 96.0)	2.83
**FIT testing (using threshold of 10 µg/g) for IBD versus non-organic pathology**					
≥50 years	75.0 (40.9 to 95.6)	70.7 (62.2 to 78.1)	14.3 (6.7 to 27.8)	97.8 (92.2 to 99.6)	2.56
18–49 years	65.0 (43.3 to 81.9)	75.8 (69.6 to 81.1)	20.3 (12.3 to 31.7)	95.8 (91.6 to 98.0)	2.68
**Organic versus non-organic GI pathology with a negative FIT (FC threshold 50 µg/g)**					
≥50 years	88.9 (56.5 to 99.4)	48.3 (38.1 to 58.6)	15.1 (7.9 to 27.1)	97.7 (87.9 to 99.9)	1.72
18–49 years	66.7 (39.1 to 86.2)	48.1 (40.5 to 55.8)	8.7 (4.5 to 16.4)	95.1 (87.9 to 98.1)	1.29
**Organic versus non-organic GI pathology with a negative FIT (FC threshold 150 µg/g)**					
≥50 years	77.8 (45.3 to 6.1)	74.7 (64.7 to 82.7)	24.1 (12.2 to 42.1)	97.0 (89.8 to 99.5)	3.08
18–49 years	41.7 (19.3 to 68.1)	78.8 (71.8 to 84.4)	12.8 (5.6 to 26.7)	94.7 (89.5 to 97.4)	1.96

FC = faecal calprotectin; FIT = faecal immunochemical test; GI = gastrointestinal; IBD = inflammatory bowel disease; NPV = negative predictive value; PPV = positive predictive value;

Increasing the FC threshold to 150 µg/g led to a lower sensitivity in both groups (86.3% [95% CI = 74.3 to 93.2] for the cohort aged 18–49 years and 81.3% [95% CI = 56.9 to 93.4] in the cohort aged ≥50 years), but specificity improved (Table 2). At this threshold, PPV was elevated to 34.7% (95% CI = 26.9 to 43.3) in the cohort aged 18–49 years, but remained low at 18.6% (95% CI = 11.2 to 29.2) in the cohort aged ≥50 years (Table 2); this reflected the higher number of IBD diagnoses in the cohort aged 18–49 years and the increased frequency of non-IBD, organic GI pathology in the older cohort.

The performance of FC to differentiate between all organic GI pathology (IBD, CRC, and other significant GI diagnoses) and non-organic GI pathology was also analysed in both cohorts; results are shown in Table 2. In patients for whom FC testing was undertaken in primary care specifically, performance was similar to that for the overall cohort. In this group, for the diagnosis of IBD versus non-organic pathology, the sensitivity was 90.9% (95% CI = 76.4 to 96.9) and the specificity 45.6% (95% CI = 39.3 to 51.3) for the cohort aged 18–49 years, and 83.3% (95% CI = 43.7 to 99.2) and 48.7% (95% CI = 40.9 to 56.6) respectively for the cohort aged ≥50 years (data not shown).

**Figure 1.**
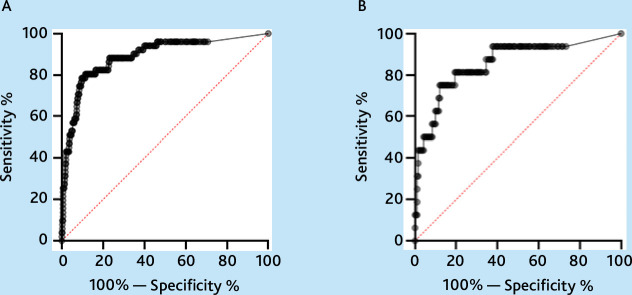
ROC curves analysing faecal calprotectin for the diagnosis of inflammatory bowel disease versus non-organic gastrointestinal pathology. A — curve analysing FC in the 18-49 age group (AUC — 0.890). B — curve analysing FC in the ≥50 group (AUC — 0.856).

ROC curves were then produced for both age groups for the diagnosis of IBD (Figure 1). For the group aged 18–49 years, the area under the curve (AUC) was 0.890 (95% CI = 0.837 to 0.943) and for the group aged ≥50 years, it was 0.856 (95% CI = 0.746 to 0.966). Using Youden’s index, an optimal cut-off for balancing sensitivity and specificity was calculated as being 263 µg/g for the group aged 18–49 years (with a sensitivity of 80.4% and a specificity of 88.6%) and 334.5 µg/g for the group aged ≥50 years (with a sensitivity of 75.0% and a specificity of 88.0%).

### FIT testing diagnostic performance

In the group aged 18–49 years, 249 patients had FIT testing performed in addition to FC. For the diagnosis of IBD, at a FIT threshold of 10 µg/g, the sensitivity was 65.0% (95% CI = 43.3 to 81.9) and the specificity 75.8% (95% CI = 69.6 to 81.1) (Table 2). In the group aged ≥50 years, using the same FIT threshold (152 patients were tested in total), the sensitivity was 75.0% (95% CI = 40.9 to 95.6) and the specificity 70.7% (95% CI = 62.2 to 78.1). For the diagnosis of organic versus non-organic GI pathology, the sensitivity was 68.4% (95% CI = 52.4 to 80.9) in the cohort aged 18–49 years, and 69% (95% CI = 50.8 to 82.7) in the cohort aged ≥50 years; specificities were 75.8 (95% CI = 69.6 to 81.1) and 70.7% (95% CI = 62.2 to 78.1) respectively (Table 2).

The performance of FC in the context of a negative FIT test was also analysed. In the cohort aged 18–49 years with a negative FIT, five out of 39 (12.8%) had an FC level of ≥150 µg/g and organic GI pathology; in the cohort aged ≥50 years, seven of 29 (24.1%) had an FC level of ≥150 µg/g and organic GI pathology (Table 2), including two with IBD. The sensitivity of FC in the cohort aged ≥50 years with a negative FIT was 88.9% at a threshold of 50 µg/g, and 77.8% at a threshold of 150 µg/g, with specificities of 48.3% and 74.7% respectively (Table 2).

### Patients with CRC

In total there were five patients who were diagnosed with CRC in the cohort aged ≥50 years. All had an FC level of >50 µg/g (Table 1), and in three of them the FC level was >150 µg/g. Four of the five patients had a FIT test before their colonoscopy; all of these tests were positive (>10 µg/g) (data not shown). Two patients in the cohort aged 18–49 years had CRC (Table 1); both had positive FIT tests and an FC level of >150 µg/g (data not shown).

### Diagnostic performance of alternative clinical markers

CRP, where tested and using a threshold of >5 mg/L, had a sensitivity of 51.1% (95% CI = 38.1 to 65.9) for diagnosing IBD versus non-organic GI pathology in the cohort aged 18–49 years, and a sensitivity of 17.3% (95% CI = 9.38 to 29.7) in the cohort aged ≥50 years (data not shown). Specificities were 77.4% (95% CI = 71.8 to 82.1) and 66.9% (95% CI = 57.9 to 74.8) respectively (data not shown).

Presenting symptoms alone were generally found to perform poorly in terms of predicting a diagnosis of IBD or organic GI pathology: the most predictive symptoms were, in the cohort aged 18–49 years, per rectum (PR) bleeding for the diagnosis of IBD, which had a sensitivity of 35.3% (95% CI = 23.6 to 49.0, *P* = 0.033) and, in the cohort aged ≥50 years, diarrhoea, which had a sensitivity of 49.1% for diagnosing organic GI pathology (95% CI = 36.4 to 61.9, *P* = 0.015) (data not shown).

Diagnostic tests and presenting symptoms that were statistically significantly associated with a diagnosis of organic GI pathology were included in multiple logistic regression models for each age group. At a threshold of 150 µg/g, FC had an odds ratio (OR) of 10.64 (95% CI = 3.04 to 43.9, *P*<0.001) in the group aged ≥50 years and an OR of 3.91 (95% CI = 1.63 to 9.64, *P* = 0.002) in the group aged 18–49 years (data not shown).

## Discussion

### Summary

These data show that FC is a sensitive test for the diagnosis of both IBD and other organic GI pathology in older and younger adults. The specificity and PPV of FC was low, however, particularly in adults aged ≥50 years at a threshold of 50 µg/g, likely driven by the higher prevalence in this group of other pathology, such as colonic polyps and diverticular disease, which are known to increase FC levels.^19,20^ Increasing the threshold at which FC is considered positive to 150 µg/g resulted in only a small improvement in PPV in the group aged ≥50 years and would potentially have missed diagnoses of CRC at this threshold if FC was relied upon as a biomarker for organic GI pathology more generally.

### Strengths and limitations

The strengths of this study are the large sample size in comparison with many other FC studies and the fact that all patients who were included had endoscopic assessment, ensuring a high reliability of the diagnoses made. The study has certain limitations, however, including the retrospective analysis of the data and the single-centre setting, which could limit the generalisability of the findings. The inclusion of patients from both primary and secondary care settings could potentially have introduced selection bias; however, the vast majority of patients (80.1%) underwent FC testing in primary care, and when testing was requested in secondary care, it was done purely for initial diagnostic purposes. There may, additionally, be a risk of bias in the selected patient cohort as inclusion criteria required both FC and colonoscopy, and the authors were unable to determine the extent to which the FC result may have influenced the decision to refer for endoscopy. Including only patients with both FC and colonoscopy, however, meant it was possible to most accurately assess the performance of FC by having accurate diagnoses made for all patients who were included in the study.

### Comparison with existing literature

One of the principal strengths of FC lies in its high NPV. The data presented here demonstrate NPVs of >97% in both age cohorts at FC thresholds of 50 µg/g and 150 µg/g, indicating that FC in the normal range in older and younger adults confers a very low probability of underlying IBD; this is in keeping with previous research by Walker *et al*,^5^ which demonstrated an NPV of 99% to distinguish functional GI disease and IBD in patients aged up to 46 years. This finding remains the case when looking more broadly at the diagnosis of organic GI pathology, rather than just IBD. However, the data presented here demonstrate a very low PPV of FC in older adults for the diagnosis of IBD; this reflects the higher incidence of other colonic pathology in older adults, such as colonic polyps and diverticular disease, which can cause elevations in FC.^21^ PPVs were also low in the group aged 18–49 years, which has previously been shown elsewhere in unselected primary care-based cohorts of patients, but it improved to a greater degree when the FC threshold was increased.^8^

This study adds to the work by Gallo *et al*, which showed that FC was able to discriminate between organic inflammatory GI disease and functional GI disorders in people aged <65 years and >65 years.^16^ Their study, however, demonstrated a higher median FC level in the older cohort (72 µg/g versus 47 µg/g), which was not the case in the study presented here. Two studies in healthy volunteers have also previously demonstrated rising FC with increases in age, but these had relatively small sample sizes.^17,22^

Although the study presented here did not evaluate patients presenting specifically with symptoms suggesting CRC, all FIT tests performed in patients who had CRC were positive; this is in keeping with data demonstrating the superiority of FIT over FC in patients with suspected CRC**.** ^23^

### Implications for research and practice

Given the rising incidence of CRC with age, it is essential to accurately stratify patients with ‘red-flag’ symptoms to ensure appropriate referral is made to exclude CRC. FIT is used in this capacity, as directed by NICE guidelines, with a threshold of 10 µg/g used to prompt endoscopic investigation.^24^ For the diagnosis of IBD and organic GI pathology more generally, FIT testing showed a good NPV for both, but lacked sensitivity compared with FC, particularly for the diagnosis of IBD; this highlights the continued role for FC, if focused on testing patients for whom there is a possibility of IBD but a low likelihood of CRC. The poor performance of symptoms alone in the detection of IBD and organic GI pathology supports non-invasive testing at initial assessment, when immediate referral is not indicated.

The use of FC in older adults could help in the stratification of patients who have a negative FIT and GI symptoms to exclude significant pathology. An elevated FC in this cohort could potentially identify those patients who would benefit from further investigations, whereas a negative FC could provide reassurance; results in the cohort aged ≥50 years that demonstrated a sensitivity of 88.9% for identifying organic GI pathology at an FC threshold of ≥50 µg/g, where FIT testing was negative, support the potential viability of this approach. However, as many of the patients in this study did not have FIT testing performed in addition to FC, further prospective data are needed to more clearly delineate the role of FC respective to FIT across different age groups.

In conclusion, the data presented here suggest that FC is a sensitive test for IBD and organic GI pathology more generally in both older and younger adults. Concerns remain over its PPV and specificity, however, particularly in adults aged ≥50 years. Although the numbers in this study cohort with CRC were small, the data highlight that FC should not be used in patients in whom CRC is suspected, when FIT and/or urgent colonoscopy are warranted. The data also suggest that increasing the FC threshold to 150 µg/g to improve its specificity (as is sometimes advocated) would result in missed CRC cases if relied on as a screening test in patients aged ≥50 years.

FC testing could have a role as a ‘rule-out’ test, providing reassurance of a low likelihood of significant organic pathology, in older adults with lower GI symptoms and a negative FIT (in whom CRC is not suspected). Prospective research in older adults to more clearly establish where FC may be positioned in diagnostic algorithms is, therefore, of great importance. Further research is also needed to evaluate the impact of FC on patient-centred outcomes, including anxiety, quality of life, and the need for further diagnostic procedures.
